# Supplementary material for: The ocular symptoms and signs during the COVID-19 pandemic
Source: PLoS One. 2022 Oct 20;17(10):e0276435. doi: 10.1371/journal.pone.0276435 (PMC9584361; doi:10.1371/journal.pone.0276435)
Supplement: Attachment — Submitted filename: Covid2022Response_220909484e.docx [file pone.0276435.s002.docx]

PONE-D-22-18069 “Possible treatment delay of dry eye during the COVID-19 pandemic: A multicenter longitudinal study” revised manuscript by Oct 21 2022.

Dear Editor,

Thank you very much for reviewing our manuscript. To aid in the review of the revised manuscript, we have included a point-by-point response to each comment.

Yours sincerely,

Kazuno Negishi, MD

Masahiko Ayaki, MD

*Additional Editor Comments:
“Both reviewers have pointed out the consideration of changing the title.”*

# We appreciate the Editor’s comment. We changed the title: “The ocular symptoms and signs during the COVID-19 pandemic: A longitudinal study”

*Reviewer’s Comments and responses*
 Reviewer #1: *The authors investigated the ocular symptoms and signs during the COVID-19 pandemic. However, it seems that this paper has gaps in its logic leading the results to the conclusions.

[Comment 1] “The authors mentioned the influence of telework has negative and positive effects on ocular symptoms and signs. There may be a lack of solid arguments or mechanisms in those effects.”*

We appreciate the reviewer’s comments. We added to the Discussion as follows.

[Discussion, page 8, line186]”Indeed, telework has negative and positive effects on ocular symptoms and signs. The negative effects include digital eye strain [12,39-41] and increased risk of DE due to increased screen time[13,14], excessive near work [11], sedentary behaviors [35], and mental stress[15]. [Salinas-Toro](https://pubmed.ncbi.nlm.nih.gov/?sort=date&term=Salinas-Toro+D&cauthor_id=34082647) et al.[12] described the high frequency of digital eye strain and dry eye disease in teleworkers during the coronavirus pandemic. The variables associated with a logistic regression model were screen time, female sex, refractive surgery, rosacea, depression, previous dry eye disease, keratoconus and blepharitis. The positive effects of telework include comfortable air conditioning in the home environment and working without face mask to avoid mask-associated DE[5-10].“

Additional References

39) Bahkir FA, Grandee SS. Impact of the COVID-19 lockdown on digital device related ocular health. Indian J Ophthalmol 2020 Nov; 68 (11):2378–2383.

40) Alabdulkader B. Effect of digital device use during COVID-19 on digital eye strain. Clin Exp Optom. 2021 Aug;104(6):698-704.

41) Ganne P, Najeeb S, Chaitanya G, et al. Digital Eye Strain Epidemic amid COVID-19 Pandemic—A Cross-sectional Survey. Ophthalmic Epidemiol. 2021 Aug;28(4):285-292.

*[Comment 2]”The word “delay,” included in the manuscript title, was not be certified logically, too.”*

We appreciate the Editor’s comments. We agree with the Reviewer that the word does not correspond to the conclusions of the study. We raised “concerns over possible treatment delays for DE during the pandemic” [Discussion, page 10, line224] and changed the title to “The ocular symptoms and signs during the COVID-19 pandemic: A longitudinal study”

Reviewer #2: *The authors investigated the patients at the first visit before and during pandemic. There were a lot of patients included that were examined with questionnaire and ocular signs. This present research was clinically informative and the method in this study to assess DE in patients was well-designed. However, a few comments should be addressed.*

*[Comment 1]”The title doesn’t fit the context of the research. The title should be clear in regard to what was found in this research. The authors shouldn’t make readers mislead.”*

We appreciate the Editor’s comments. We changed the title to “The ocular symptoms and signs during the COVID-19 pandemic: A longitudinal study”

*[Comment 2]”This is not a multi-center study. The study included only two institute.”*

We appreciate the Editor’s comments. We deleted “multi-center” in title and line 79.

*[Comment 3]”Did the author perform the test for normal distribution?”*

We appreciate the reviewer’s comments. We performed Kolmogorov-Smirnov Test to check the normal distribution of age and it was not normally distributed in three groups. Accordingly, we recalculated and amended the Methods and footnote of Table 1 as follows.

[Methods, page 5, line 120] “Mann Whitney U test with Bonferroni correction”

[Footnote of Table 1]”*P<0.05, calculated by Mann Whitney U test with Bonferroni correction and chi square test”

*[Comment 4]”Please describe more in detail about how the authors perform the regression analysis in association with the pandemic. Which data did you use on this analysis and how did you analyze?*

We appreciate the reviewer’s comments. We amended the text as follows.

[Methods, page 5, line 121]”To identify which ophthalmic parameters were correlated with the pandemic, regression analysis was performed using the partial correlation coefficient, with the year 2019 (before the pandemic) and the year 2020 (beginning of pandemic) as dependent variables, while corneal parameters (BUT, strip meniscometry, Schirmer test, and corneal staining), diagnosed DE, and refractive error were used as independent variables.”

[Footnote of Table 2] “Before the pandemic (year 2019) = 0, during the pandemic (year 2020) = 1; short BUT = 1, normal = 0; low strip meniscometry value =1, normal = 0; low Schirmer test value = 1, normal = 0; positive corneal staining = 1, negative = 0. Each cut-off value is specified in Table 1.”
