# Supplementary material for: The ocular symptoms and signs during the COVID-19 pandemic
Source: PLoS One. 2022 Oct 20;17(10):e0276435. doi: 10.1371/journal.pone.0276435 (PMC9584361; doi:10.1371/journal.pone.0276435)
Supplement: Attachment — Submitted filename: Covid2022ResponseR2.docx [file pone.0276435.s003.docx]

Dear Editor,

Thank you very much for reviewing our manuscript. To aid in the review of the revised manuscript, we have included a point-by-point response to each comment.

Yours sincerely,

Kazuno Negishi, MD

Masahiko Ayaki, MD

Reviewer’s Comments and responses *Reviewer #1: [Comment 1] “Line 40: The word “should” may not be suitable. I recommend “might” or more weakened wordings.”*

We changed the word, accordingly.

[Abstract, Page2, Line39]” It might be considered”

*[Comment 2] “Line 74: Delete the word “longitudinal.” Longitudinal study must contain repeated observations of the same patients. In this study, the identicalness was not certified during periods.”
 [Comment 3] “Line 80: Delete the word “longitudinal.””*

We deleted the word “longitudinal” in the title, line57, line73, and line79, accordingly.

*[Comment 4] “Line 208-213: Delete the paragraph. This paragraph is not directly associated with the substance of this study, and encourage the overestimation.”*

We deleted the paragraph.
